# Lytic granule exocytosis at immune synapses: lessons from neuronal synapses

**DOI:** 10.3389/fimmu.2023.1177670

**Published:** 2023-05-18

**Authors:** Hsin-Fang Chang, Claudia Schirra, Varsha Pattu, Elmar Krause, Ute Becherer

**Affiliations:** Department of Cellular Neurophysiology, Center for Integrative Physiology and Molecular Medicine (CIPMM), Saarland University, Homburg, Germany

**Keywords:** synapse, CD8+ cells, cytotoxic T lymphocytes, neuron, exocytosis, endocytosis, SNARE proteins

## Abstract

Regulated exocytosis is a central mechanism of cellular communication. It is not only the basis for neurotransmission and hormone release, but also plays an important role in the immune system for the release of cytokines and cytotoxic molecules. In cytotoxic T lymphocytes (CTLs), the formation of the immunological synapse is required for the delivery of the cytotoxic substances such as granzymes and perforin, which are stored in lytic granules and released *via* exocytosis. The molecular mechanisms of their fusion with the plasma membrane are only partially understood. In this review, we discuss the molecular players involved in the regulated exocytosis of CTL, highlighting the parallels and differences to neuronal synaptic transmission. Additionally, we examine the strengths and weaknesses of both systems to study exocytosis.

## 1 Introduction

**Figure 1:**
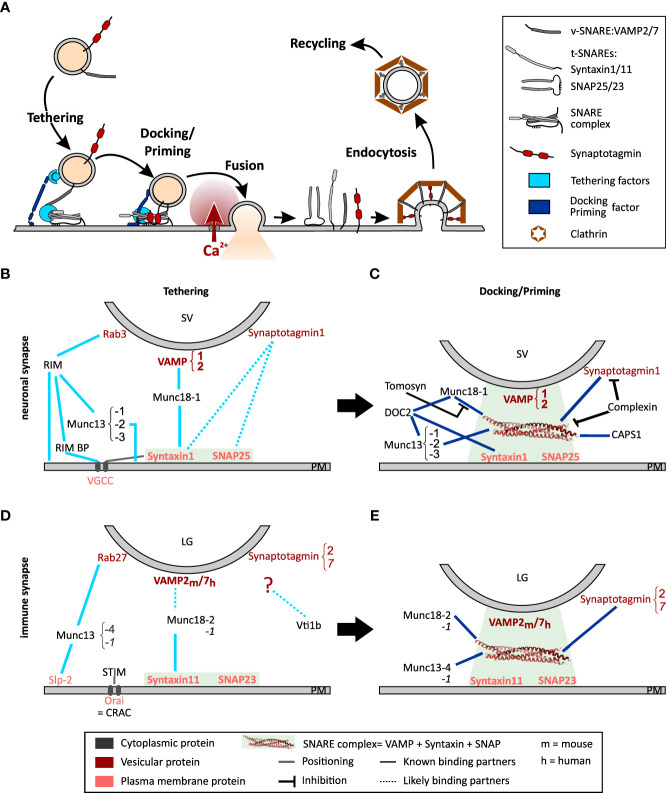
Model of the exocytosis machinery. **(A)** Schematic representation of the steps that SVs and LGs undergo before and after fusion with the plasma membrane. For sake of clarity only selected proteins involved in this process are shown. More comprehensive protein-protein interaction networks are shown in **(B**–**E). (B, C)** Protein Interactions occurring during tethering **(B)** and docking/priming **(C)** of SVs in neurons. **(D, E)** Protein interaction required for tethering **(D)** and docking/priming **(E)** of LGs in CTL. Bottom legends applies to panels **(B-E)** The light green squares indicate the strong interaction of the t-SNAREs during tethering and of the entire SNARE complex during docking/priming. Lines indicate protein interactions, stippled lines show probable protein interactions. Black lines with blunt arrow correspond to inhibitory interactions.

CD8+ cytotoxic T lymphocytes (CTLs) eliminate virus-infected or cancerous cells by releasing pore-forming perforin and proteases such as granzymes that induce apoptosis of target cells. These toxic substances are stored in lytic granules (LG), which undergo exocytosis upon T cell antigen receptor (TCR) signaling, releasing cytotoxic molecules at the contact zone with the target cells. This contact zone, where TCR-mediated signal transduction and secretory events take place, is named the immunological synapse (IS) (1). Exocytosis of LGs is a highly regulated process ensuring that the cytotoxins are delivered only to the target cell. LG exocytosis requires tethering, docking/priming and finally fusion with the plasma membrane. After exocytosis, the membrane of the granule is retrieved and recycled (**Figure 1A**). These steps are tightly controlled by a complex molecular machinery.

Interestingly, very similar mechanisms govern neuronal synaptic transmission. The release of neurotransmitters by neurons is by far the most highly regulated form of exocytosis known. The speed, accuracy and temporal resolution found at synapses are unmatched. Due to their complexity and importance, the mechanisms involved have been the target of intense study. A variety of the molecules discovered at neuronal synapses have recently been found to have similar roles in LG exocytosis. Furthermore, a number of immunological deficits have been tied to mutations of proteins involved in neuronal synaptic transmission. The mechanisms underlying LG exocytosis in CTLs are not well characterized and their understanding has benefited greatly from the knowledge of synaptic transmission. The aim of this review is to describe the molecular process of neuronal synaptic vesicle release in neurons and compare it with that of LG exocytosis in CTLs. We highlight the similarities and differences between the two systems and identify gaps in our current understanding of these key cellular processes.

## 2 Regulated exocytosis in neurons

The core molecular machinery for membrane fusion is formed by the soluble N-ethylmaleimide-sensitive factor attachment receptor (SNARE) proteins. They were discovered by R. Schekman in yeast (2) and J. Rothman showed that these proteins also exist in mammals where they perform similar tasks (3–5). Subsequently, their function was elucidated in neurons, by studying the effects of proteolytic clostridial neurotoxins, i.e. tetanus- and botulinum-toxins. These toxins specifically cleave SNARE proteins leading to a complete arrest of neurotransmitter release (6, 7). The resulting work demonstrated that the SNARE complex is a fusion machine, which provides the force that drives the fusion of synaptic vesicles (SV) with the pre-synaptic target membrane. The SNARE complex consists of a vesicular SNARE (v-SNARE), synaptobrevin, and two target SNAREs (t-SNAREs), syntaxin-1 and synaptosomal-associated protein 25 (SNAP-25), located on the plasma membrane. Synaptobrevin and syntaxin-1 contain one SNARE motif, while SNAP25 contains two. These coiled-coil motifs assemble into tight four-helix bundles called “trans”-SNARE complexes, which attach the SV to the presynaptic membrane. Induction of SV fusion with the plasma membrane requires the assistance of many other proteins in a well-coordinated fashion, as described thereafter.

### 2.1 The SV fate: from the reserve pool to fusion

The molecular mechanism of synaptic vesicle exocytosis is highly complex and is described in detail in excellent reviews (8–11). Therefore, it will not be repeated in this review. Here, we present an outline of the molecular events leading to SV fusion.

SV are maintained in a reserve pool by the mesh-forming synapsin (12). Under activation by Ca^2+^-calmodulin, this mesh dissolves releasing SVs that move toward the active zone at the pre-synapse along F-actin using myosin II or V as the motor protein (13–15). An overload of SV at the active zone is prevented by a dense cortical F-actin meshwork that functions as a semipermeable barrier, which dynamically regulates plasma membrane accessibility (16–18). This role of F-actin has been studied in great detail in neuroendocrine cells (see Meunier and Gutierrez (19) for review). Upon arrival at the active zone, SVs loosely attach to the plasma membrane by means of a tethering mechanism consisting of multilayered protein interactions (**Figures 1A, B**). The first tether consists of the active zone proteins RIM, RIM-binding protein, and Munc13-1 or Munc13-2, which attach to the SV *via* Rab3 (20–22). A second tether is generated by Munc18-1, which bridges the t-SNARE, syntaxin-1, and the v-SNARE, synaptobrevin2 (23, 24). Finally, it was proposed that synaptotagmin1 interacts directly with syntaxin-1 and SNAP25 thereby pulling the SV closer to the plasma membrane. However, while this last tether has been reported to attach secretory vesicles to the plasma membrane in neuroendocrine cells (25), its relevance at neuronal synapses is still under debate (26). SV proximity to voltage dependent Ca^2+^ channels (VGCC) is promoted by the t-SNAREs and Munc18-1 (27–29).

At this stage the SVs can be docked, i.e. primed. Nowadays, both terms describe the same step but they have been defined by different techniques. While docked SVs are defined by their direct contact with the plasma membrane in electron micrographs, priming of a vesicle corresponds to the ability of the SV to fuse with the plasma membrane as measured by functional assays (see 2.3). As the resolution of electron microscopy has improved, the analysis of electron micrographs has become more precise, and it is now accepted that docking equates to priming, which is why we refer to this process as docking/priming (30). For docking/priming, the v-SNARE synaptobrevin2 interacts with the t-SNAREs, SNAP25 and syntaxin-1 in a Munc18-1 and Munc13-1 or -2 dependent manner (**Figures 1A, C**). Indeed, while Munc18-1 forms a template for the SNARE complex, Munc13-1 or -2 is required to open syntaxin-1 allowing its interaction with SNAP25 and synaptobrevin2 (31–33). This corresponds to a loose docking/priming step in which the SNARE complex is only partially formed. Now synaptotagmin 1 that is partially bound to syntaxin-1, attaches to the entire SNARE complex, which is stabilized by complexin (34, 35) (**Figures 1A, C**). Finally, a steep increase of the presynaptic intracellular Ca^2+^ concentration, due to the opening of the VGCCs, induces the complete zippering of the SNARE complex and the dipping of the C2A domain of synaptotagmin 1 in the plasma membrane (36–38). Both actions pull the vesicle membrane close enough to the plasma membrane to induce fusion. Immediately after fusion the SNARE complex is disassembled by N-ethylmaleimide-sensitive factor (NSF) through its ATPase activity, with the help of SNAPs to allow v-SNARE recycling (39–41).

One should notice that SV docking/priming is assisted by several additional priming factors (**Figure 1C**). The Ca^2+^-dependent activator protein for secretion-1 (CAPS1) promotes priming *via* interaction with syntaxin-1 probably downstream of Munc13-1 or -2 (42, 43). Similarly, DOC2A and DOC2B interact with syntaxin-1, Munc18-1 and Munc13-1 or -2 to promote exocytosis in a phorbol ester- and Ca^2+^-dependent manner (reviewed by Pinheiro et al. (44)). Finally, snapin binds to synaptotagmin enhancing its interaction with the SNARE complex, thereby stabilizing SV priming and enhancing exocytosis at low intracellular Ca^2+^ concentration (45–47). Few proteins inhibit docking/priming. The most prominent is probably tomosyn (STXBP5) that competes with Munc18-1 for its interaction with syntaxin-1 (48) and additionally binds to SNAP25 thereby forming a dead-end tomosyn-SNARE complex (49, 50).

### 2.2 Vesicle pools and recycling

In order to allow for a sustained high rate of neurotransmitter release, each of the steps leading to SV fusion is carried out simultaneously by numerous synaptic vesicles. These form individual pools of tethered vesicles and/or docked/primed vesicles ready for exocytosis. This ensures that docked/primed vesicles are released within 1 ms after depolarization of the pre-synapse. However, since primed vesicles are fully release-ready, they must be prevented from fusing. This task is performed by complexin, which not only stabilizes the fusion machinery but also has a regulatory function (34, 35). Furthermore, the tethered and the reserve pools allow a steady replenishment of readily releasable vesicles (51–53). A tight coupling between exo- and endocytosis ensures maintenance of these pools to sustain neurotransmitter release, and homeostasis of membrane composition (54–56). In so-called kiss-and-run exocytosis, the fusion of SVs with the plasma membrane is transient and the membrane of the vesicle does not mix with the plasma membrane (57, 58). On the contrary, in the full fusion mode, the SV membrane completely integrates in the plasma membrane. An ATP dependent dynamic assembly of filamentous actin, involving N-WASP and formin, appears to be required for this type of fusion (59). The membrane components of the SVs are recycled *via* classical clathrin-mediated endocytosis, fast endocytosis or bulk endocytosis. The mode of endocytosis depends on the strength of the stimulus, i.e. the pre-synaptic Ca^2+^ concentration, and the amount and the speed of SV fusion (60, 61). Finally, SV filling with neurotransmitter occurs on site *via* specific transmembrane transporters.

### 2.3 Main methods to study neurotransmission

This complex model of the exocytosis machinery at synapses was resolved through the combination of a wide array of techniques (**Table 1**). With biochemical techniques it was possible to define the minimum fusion machinery and to tease out direct molecular interactions. The core of these techniques involved *in-vitro* lipid mixing assays in which donor and acceptor artificial membranes are reconstituted with a variety of lipids and proteins and their fusion kinetics are analyzed [reviewed in Grothe et al. (62)]. Co-immunoprecipitation assays of the proteins involved in exocytosis coupled with site specific mutagenesis allowed precise determination of protein domain function. Additionally, crystallography of the full SNARE complex alone or in association with other proteins such as Munc18-1 have revealed in detail the amino acids involved in these inter-protein interactions [reviewed in Brunger et al. (63)]. The results obtained with these techniques were validated in the cellular environment by testing the effects of gene deletion or mutation on synaptic transmission. The classical approach to detect synaptic transmission is the measurement of postsynaptic currents in patch clamp experiments. This has provided the basis for our current understanding of exocytosis. Alternatively, the fusion of individual vesicles with the plasma membrane can be precisely assessed with high temporal resolution by membrane capacitance measurements (64, 65). However, this method can be applied only to very large synapses in very demanding experiments (66, 67). Therefore, almost all these experiments were performed on neuroendocrine cells (i.e. chromaffin cells) and the results were extrapolated to neurons (68–70). In addition, total internal fluorescence microscopy (TIRFM), which can visualize fluorescently labeled vesicles prior to and during release, allowed for more detailed assessment of tethering and docking/priming (71–73). In parallel, the impact of genetic modifications on the ultrastructure of synapses, i.e. on different vesicle pools, was analyzed by electron microscopy [(26, 74, 75); for review see Zuber and Lučić (76)]. One remaining problem was to understand how the proteins involved in exocytosis are organized at the pre-synapse. This was largely solved with the advent of super-resolution microscopy, such as stimulated emission depletion (STED) microscopy and direct stochastic optical reconstruction microscopy (dSTORM)/photo-activated localization microscopy (PALM) (77, 78), and CLEM experiments, in which super-resolution microscopy is combined with electron microscopy (EM) (79). Finally, live cell imaging of fluorescent markers allowed differentiation between events occurring during exocytosis, endocytosis or vesicle recycling. In early experiments, endocytosis was visualized using fluorescent dyes such as FM1-43, which are taken up in an activity dependent manner (80). Later, these experiments were performed with pH sensitive GFPs such as the super ecliptic pHluorin [SEP, Miesenbock et al. (81)] or RFPs, like pHuji (82), linked to the luminal domain of a SV protein such as synaptophysin [SypHy; Granseth et al. (83)]. In inactive synapses the fluorophore is located in the acidic SV lumen and is therefore quenched. Upon exocytosis the fluorescent protein is exposed to the neutral extracellular medium inducing a strong increase of its fluorescence. Upon endocytosis the pH sensitive fluorescent protein is re-internalized and quenched again by re-acidification of the SV lumen. As a result neuronal activity is visualized by fluorescence intensity variation at the synapses (84, 85).

**Table 1:** Methods to analyze exocytosis.

Experimental methods		Gain of knowledge	Cell type; Application	
			Neurons	CTLs
Functional assays				
Electrophysiology	Synaptic transmission	Transmitter release	Used extensively; Easy to implement.	NA
	Membrane capacitance	Vesicle fusion	Complex; Restricted to large synapse	Complex, restricted to human CTL
Live cell imaging	Fluorophore uptake (for example FM1-43). Wide field fluorescence microscopy.	Endo- and exocytosis	Relatively easy to implement	Use with caution as it also reports phospholipid scrambling
	Overexpression of pH sensitive fluorescent protein tagging a vesicular protein. Wide field or TIRF microscopy.	Labelling of vesicular content.		
		Content release and pre-fusion steps	NA for neurons but used in neuroendocrine cells	Labelling of granzyme B or perforin. pH sensitivity of fluorophore is not mandatory
		Labelling of a vesicular membrane protein. The fluorescent label is directed toward the vesicle lumen		
		Exo- endocytosis and prefusion steps	Tagged proteins: synaptophysin and synaptobrevin. Tag is mainly SEP. Used extensively. Easy to implement	Tagged protein: synaptobrevin. Tag are SEP or pHuji. Used extensively. Easy to implement
	Fluorescent antibody uptake. Antibody is directed against the luminal part of a vesicular membrane protein or to a tag directed toward the lumen of the vesicle.	Endocytosis	Easy to implement: Used with antibody against synaptotagmin1	Easy to implement: Used in combination with the expression of synaptobrevin-RFP. Antibody against the fluorescent protein.
Killing assay	Various techniques exist in which lysis or apoptosis of target cells are measured.	Release of content; Assessment of its toxicity	NA	Staining of cell death. Applicable for live and fixed cells, depending on the selected method
Flow cytometry	Degranulation assay based on LAMP1 recycling	Exocytosis	NA	Easy to use High throughput method
ELISA	Measure released protein activity	Release of content	NA	Easy to use. Commercially available. i.e. Granzyme B activity kit.
Structural information				
Light microscopy	Colocalization experiments with super resolution microscopy	Localization of the exocytotic machinery	Performed with STED or dSTORM/PALM	Performed with SIM or confocal microscopy
Electron microscopy	TEM, SEM and 3D tomography (i.e. FIB-SEM)	Ultrastructure of the synapse	Size of vesicle pools. Localization of endocytosis and recycling vesicles.	Organization of the IS. Localization of exo- endocytosis and recycling vesicles. Visualization of content release (SMAPs and extracellular vesicles)
	CLEM or immunogold labelling	Localization of proteins at the synapse	Applicable for both system in nearly the same manner.	
Biochemical assays				
Lipid mixing assays	Reconstitution of donor and acceptor membranes containing different fluorophores. Mixing measured by FRET	Reconstitution of fusion machinery. Fusion kinetics. Identification of protein domain function.	Identification of cognate SNAREs. Measurement of tethering and priming factor activity.	Measurement of priming factor activity.
Immuno-precipitation assay	Co-immunoprecipitation (also yeast two hybrid)	Identification of interaction partners	Applicable for both system in the same manner.	
	Immuno-isolation after subcellular fractionation	Organelle identification and purification	Applicable for both system in the same manner.	

Note that all these methods can be combined with molecular biology methods to study the effect of mutations in proteins involved in exo- or endocytosis. NA refers to not-applicable.

Taken together, the last 25 years of intensive study of synaptic transmission have revealed in minute detail the components of the exocytotic machinery and the precise timing of protein interactions required for SV exocytosis. These studies of neuronal synapses preceded the description of the IS and resulted in key concepts of the molecular mechanisms of synaptic vesicle docking/priming that appear to be valid for other cellular models. We will now discuss how they can be adapted to describe LG exocytosis at the IS of CTLs.

## 3 Regulated exocytosis in cytotoxic T cells

How can this detailed knowledge of neurotransmission help us understand LG release at the IS? CTLs are part of the adaptive immune system. They circulate in the blood stream and patrol tissues and organs to detect infected cells and tumor cells. When CTLs detect a target cell for which they express a specific T cell receptor, they form a synaptic interface, i.e. the IS, with the target cell, and deliver cytotoxic molecules to the synaptic cleft *via* fusion of LG with their plasma membrane. This process is largely identical in CTL and natural killer (NK) cells. The main difference between the two cell types lies in the recognition of the target cells. For this reason, we will discuss the mechanisms of LG exocytosis using data obtained on both cells (86). The release of perforin and granzymes is a highly regulated process that is essential to kill the infected or malignant target cell. Indeed, loss of cytotoxicity in CTLs and NK cells results in an immune deficit, as is the case for the human immune disease, hemophagocytic lymphohistiocytosis (HLH) and familial hemophagocytic lymphohistiocytosis (FHL).

### 3.1 How a human disease helped to solve LG fusion mechanisms

The first indications that LG exocytosis shared similar features with SV exocytosis did not come from well-planned experiments but rather from clinical observations in patients affected by clinical syndromes such as FHL and HLH, which lead to reduced or abolished cytotoxicity of CTL and NK cells. The underlying causes of these defects are: 1. CTLs or NK cells lack or express a mutated form of the cytotoxic protein perforin (*PRF1*) causing FHL type 2 (87); 2. their cytokine production is impaired due to the mutations of either *SH2D1A/SAP* (coding for SLAM-associated protein), *ITK* (Tyrosine protein kinase) or *CD27* (receptor for TNF) inducing FHL type 1 (88–90); 3. the biogenesis of LG is perturbed (gene mutation of *LYST*, *AP3B1* or *XIAP/BIRC4*) (91–93); 4. Immune cells are unable to release LG content due to defective exocytotic machinery (94, 95). In the latter case, the mutated proteins include the SNARE protein, syntaxin11 (*STX11*, FHL 4) (96), and three tethering/priming factors Rab27a (*RAB27A*, Griscelli syndrome type 2), Munc13-4 (*UNC13D*, FHL 3) and Munc18-2 (*STXBP2*, FHL 5) (97–100). All these proteins are the same or isoforms of proteins involved in SV exocytosis. By the time they were discovered in CTLs, their function was elucidated to a large degree in neurons. Hence these results not only sparked the interest of immunologists but also of neuroscientists who had access to a large array of genetically modified mice in which these and other genes involved in exocytosis are deleted. Their interdisciplinary collaboration generated considerable advances in the understanding of LG release in CTLs and NK cells.

### 3.2 Methods to investigate LG exocytosis

The description of cell biological processes in immunology has historically been performed with a different repertoire of methods allowing high cell throughput (**Table 1**). Flow cytometry is of great importance for the characterization of heterogeneous cell populations but also for analyzing the expression of cell surface and intracellular molecules, and for the detection of cellular processes, such as exocytosis. A standard method for analyzing the LG exocytotic rate is the degranulation assay, which is based on flow cytometry. This method allows one to quantify the uptake of fluorescence-labeled antibodies raised against the intraluminal domain of lysosome-associated membrane protein 1 (LAMP-1, also named CD107a). When LG fuse with the plasma membrane, the intraluminal domain of LAMP-1 is exposed to the extracellular medium, and the anti-LAMP1 antibody contained in the medium can bind to it (101, 102). Hence, the brightness of the cell is then directly proportional to the number of exocytosed vesicles. This very potent method has been widely used to study LG exocytosis in CTL and NK cells from HLH or other immune-deficient patients (103). This flow cytometry based high throughput analysis generates solid results in a timely manner as thousands of cells can be screened rapidly. However, obtained results need to be interpreted with caution since LGs are only a fraction of the lysosomal compartments, all of which contain LAMP1 (104, 105). Population based functional assays should be used to complement the degranulation assay. One of these approaches is the killing assay, which measures the ability of CTL to kill target cells by quantifying signals from lysed target cells, such as lactate dehydrogenase release, surface phosphatidylserine expression, propidium iodide uptake or decay of fluorescence of intracellular dyes (106–109). The interpretation of these experiments must also take into account the ability of the CTL to kill *via* Fas-FasL that occurs *via* a different vesicular trafficking pathway (110).

While these methods were sufficient to identify proteins that are involved in LG release, they do not allow determination of their function during exocytosis. This limitation was solved *via* high resolution single cell imaging techniques such as confocal microscopy or TIRFM that allow the visualization of LG exocytosis in real time (104, 111, 112). In particular, TIRFM enabled the investigation of IS formation, docking/priming of LGs and their fusion kinetics. In these experiments the cells are seeded on glass slides or on supported lipid bilayers that display adhesion molecules and cognate peptide in the major histocompatibility complex (pMHC) or anti-CD3 antibody that triggers IS formation (113–116). The LGs are labeled with Lysotracker or more specifically *via* the expression an LG protein bound to a fluorescent protein. For example granzyme B-mTFP was used to monitor the release and diffusion of the LG content, while synaptobrevin2-mRFP allows observation of the fate of the fused LG membrane in the plasma membrane (104). In addition, using a pH sensitive label such as SEP or pHuji enables the precise measurement of the LG with the plasma membrane, fusion time, i.e. of the pore opening for the release of lytic components (81, 82). To obtain more detailed information about LG size and fusion kinetics, membrane capacitance measurement using patch-clamp electrophysiology was applied (117). However, adapting patch clamp electrophysiology to primary human CTLs has been extremely challenging and almost impossible for mouse CTLs. Hence this method will have limited use in the future. Live cell methods were complemented by the analysis of the IS ultrastructure with electron microscopy and CLEM (111, 118, 119). Finally, biochemical assays, such as lipid mixing assays, co-immunoprecipitation assays, or crystallography have been used to better understand the intrinsic properties of each protein involved in LG release (120).

### 3.3 Fate of LGs from IS formation to fusion

In contrast to neurons, the IS is not a long-lived structure in which vesicles are poised to exocytose. As a correlate the LGs are not organized in vesicle pools and the steps upstream from exocytosis are somewhat different in CTLs as compared to neurons. However, the overall sequence of events is similar, including the final transport of LG to the plasma membrane, the tethering and the docking/priming steps. The proteins mediating these steps are either identical or they are highly conserved paralogs from those proteins involved in synaptic transmission in neurons.

The IS is formed on demand upon recognition by the CTL through the TCR of the antigenic peptide associated to the major histocompatibility complex (pMHC) (1, 121). In NK cells, IS formation with the target cell is initiated by the combination of two signals. The first is a lack of MHC1 recognition (disinhibition), and the second is a positive signal from a variety of germline-encoded activation receptors that bind to proteins such as lectins or hemagglutinins on the target cell (122). Target cell recognition then triggers complex signaling cascade that leads to a rapid realignment of the Golgi complex and microtubule network by shifting the microtubule organizing center (MTOC) toward the IS and polymerizing microtubules toward the distal pole of the cell. Along these microtubule tracks, LGs and a variety of other organelles, such as multi vesicular bodies and mitochondria, move toward the IS. This function of the MTOC is important to ensure LG delivery to the IS but not for their exocytosis as such (123). Once close to the plasma membrane LGs switch their transport pathway from tubulin to F-actin through myosin IIa (124, 125), which is reminiscent of the transport of SVs to the active zone. Their final destination is the secretory domain of the central-supramolecular activation complex (c-SMAC). Similar to neurons, in CTLs actin also appears to form a barrier for LGs that prevents them from joining the IS. In fact, actin clearance at the c-SMAC is required for LG exocytosis. (112, 126). Thus, a fine balance in the density of the F-actin network appears to be required for LG secretion to occur (127).

Concurrent with LG transport, the plasma membrane at the IS adapts to become a platform for LG fusion. One of the modifications consist of an accumulation of Orai Ca^2+^ channels, that occurs simultaneously to an IP_3_/Ca^2+^ dependent activation and translocation of STIM proteins to the ER close to the IS. Activated STIM proteins interact with Orai, forming the store-operated Ca^2+^ release activated Ca^2+^ (CRAC) channel complex, which open leading to store operated Ca^2+^ entry (SOCE) (128–130). The resulting increase in cytoplasmic Ca^2+^ concentration is enhanced by nearby mitochondria (131, 132) ensuring synaptic activation (133, 134). The second modification ensures that the molecular components of the release machinery are at the right place. For that the t-SNARE syntaxin11, which is required for LG fusion, translocates to the IS and integrates into the plasma membrane in a VAMP8 dependent manner (135, 136). Membrane patches with syntaxin11 accumulation serve as hotspots for LG release. Whether SNAP23, the second t-SNARE, relocates to the plasma membrane during IS formation is not known.

At this stage the IS is ready for LGs to tether to the plasma membrane *via* two different protein complexes. The first is composed of Rab27a that associates with LG membranes in a GTP dependent fashion, the synaptotagmin like protein-2 (Slp-2) anchored in the plasma membrane and probably Munc13-4 (121, 137–139) (**Figure 1D**). The second consists of syntaxin11 at the plasma membrane and Munc18-2 as a bridge to the LGs (140). The identity of the LG protein to which Munc18-2 binds at this stage is elusive. By analogy to neuronal tethering of SVs, we speculate that it is a v-SNARE. This tether is likely the gateway for docking/priming in which SNARE complex assembly is initiated (**Figure 1E**). As indicated above, the t-SNAREs forming this complex are syntaxin11 (135, 141) and SNAP23 (96, 142), while the v-SNARE is VAMP2 in mouse and VAMP7 in human CTLs (118, 142). The SNARE complex assembly is mediated by Munc18-2 and Munc13-4. Similarly to the role of Munc18-1 in neurons, Munc18-2 is required in CTLs as a chaperone for syntaxin11 (140) and favors SNARE complex formation (120). The role of Munc13-4 is still under debate. Early studies showed that it is involved in a step prior to exocytosis in which recycling and late endosomal vesicles fuse to form LGs (143). More recent work demonstrated that Munc13-4 is required for priming (144). Whether Munc13-4 is required for opening syntaxin11 prior to SNARE complex formation is likely but not fully elucidated (145). Interestingly, the neuronal Munc18-1 and Munc13-1 are also expressed in human and mouse CTLs. Both can sustain LG exocytosis and they appear to have a compensatory role when Munc18-2 and Munc13-4 are deficient (140, 144, 146). Finally, upon increased Ca^2+^ concentration, the SNARE complex fully zippers and LGs fuse with the plasma membrane releasing perforin and granzymes into the synaptic cleft. The SNARE complex disassembly following fusion that is required for LG membrane recycling has not been investigated until now in CTL nor in NK cells.

The Ca^2+^-dependence of LG exocytosis has been described in many excellent reviews, so we will not address it here. Instead, we will discuss the identity of the Ca^2+^ sensor that assists the zippering of the SNARE complex. Mouse CTL express synaptotagmin 2, 7, 11 and 16 but only synaptotagmin 2 and 7 are Ca^2+^ sensitive (147). While synaptotagmin 7 is undoubtedly involved in LG exocytosis whether it is the Ca^2+^ sensor that triggers LG fusion with the plasma membrane is under debate (147–149). In fact, Sleiman et al. (147) showed that in mouse CTLs synaptotagmin 7 is rather involved in LG trafficking whereas synaptotagmin 2 might be the actual Ca^2+^ sensor for secretion. An alternative candidate is Munc13-4 as it contains Ca^2+^ binding N- and C-terminal C2 domains (C2A and C2B). Mutations of these C2 domains that alter their Ca^2+^ sensitivity, abolished LG exocytosis in NK cells (150). While the C2A domain participates in SNARE complex zippering, the C2B binds to the lipids of the plasma membrane upon increased Ca^2+^ concentration (145) possibly leading to the final pull on the vesicle membrane to induce fusion. Interestingly, in neurons Munc13-1 also plays an important role in calcium sensing albeit for a different function namely the replenishment of the RRP (151, 152).

Overall, the major steps that LGs undergo before fusing with the plasma membrane are the same as those that SVs must undergo for synaptic transmission. However, the timing of LG exocytosis is not as precise as SV in neurons. Whereas the latter occurs within milliseconds of the Ca^2+^ trigger, LG exocytosis takes minutes. Therefore, a smaller number of proteins seems to be required to control LG exocytosis.

### 3.4 Kinetic of fusion and content release

As for SVs, full fusion and kiss-and-run/kiss-and-stay modes can be detected for LG secretion in NK cells (153) while only full fusion was reported in CTL until now (105, 115). Furthermore, the fusion kinetics of full fusion events can vary. Estl et al. (105) found that LG stained through the expression of synaptobrevin2-pHuji showed a fluorescent signal that disappeared after fusion with the plasma membrane according to two different time courses. In 80% of the cases LG fluorescence disappeared in less than a second (300 ms in average, at 20°C). In the remaining 20%, fluorescence decay was much slower with an average time of 308 s. These two different membrane mixing behaviors might be explained by fast *vs.* very slow fusion pore dilation or by stable clustering of synaptobrevin2 in the plasma membrane at the fusion site. It will be interesting to elucidate whether F-actin, as in neuroendocrine cells, is involved in the expansion of LGs fusion pores (59). In contrast, all exocytosis events that were analyzed, showed a content release (labelled *via* granzyme B-mTFP expression) in about 300 ms. Events, in which content diffusion was much slower, were ignored from the analysis as it was questioned whether they corresponded to proper LG exocytosis. However, in a groundbreaking work, Bálint et al. (154) decisively expanded the view of LG exocytosis. They showed that granzyme B can be released as a soluble protein or within previously overlooked particles. The insoluble granzyme B particles were coined supramolecular attack particles (SMAPs). Subsequently, Chang et al. (119) showed that LGs can be divided in two different populations. While SMAPs were localized to multicore granules, diffusible granzyme B was found in classical single core lytic granules. The SNAREs involved in release of both types of LGs are probably identical because both harbor synaptobrevin2 on their surface (119). Whether tethering or priming factors are specific for each type of granule remains to be established.

### 3.5 LG recycling

CTLs actively move from one target cell to another, eventually killing a large numbers of target cells within minutes to hours. Killing one target cell, which requires the release of only few LGs, does not appear to need efficient LG recycling. However, this is not the case for CTLs that engage in serial/simultaneous killing of multiple target cells (155–157). Accordingly, the importance of LG endocytosis and recycling to sustain molecular signaling has been demonstrated (157, 158). The endocytic pathway was unraveled by following the clathrin- and dynamin-dependent endocytosis of the LG membrane protein synaptobrevin2. Like in neurons and neuroendocrine cells, LG endocytosis is promoted by the F-actin MyosinII complex (16, 159). Re-acidification of the endocytosed vesicle occurred within two minutes at 37°C (157). They were then recycled through early and late endosomes where they were refilled with granzyme B, and most likely with all other LG components, such as perforin and serglycin, to generate fully functional LGs. The endocytosed synaptobrevin2 containing granules rapidly mature by acidification of their lumen and reacquisition of cytotoxic proteins *via* late endosomes. Full recycling of LGs requires 30 to 60 minutes in mouse CTLs before they can be used for the next round of exocytosis (157). *De-novo* synthesis of key cytotoxic proteins perforin and granzymes in addition to interferon gamma and TNF-alpha is supported by mitochondria. In fact their depletion resulted in a significant reduction in the serial killing ability of CTLs (160). In NK92 cells some lytic components of the LGs, such as granzyme B and perforin, can be captured after exocytosis, recycled and reused for a second round of exocytosis, contributing to NK cell cytotoxicity (161, 162). Finally, as in neurons the coupling between LG exo- and endocytosis is mediated by Ca^2+^. In this context the calcium channel flower domain-containing protein 1, in short Flower, plays a major function (163). Flower deficient CTLs display a time-dependent block of LG endocytosis that is rescued by reintroduction of Flower in CTLs or by raising the extracellular Ca^2+^ concentration. Interestingly, a similar role for Flower has been demonstrated in neurons (164).

These findings show how CTLs, upon TCR triggering, combine several mechanisms, such as tight coupling of LG exo- endocytosis and mitochondrial-dependent cytotoxic protein translation, to maintain a constant supply of LGs during killing. This may be one of the many ways in which CTLs achieve a high per-capita killing capacity and therefore function as efficient serial killers (165). This feature may have tremendous importance *in vivo* for efficient clearance of tumors and viral infections.

## 4 Differences of LG fusion machinery compared to neurotransmission

Our understanding of LG exocytosis in CTLs has increased rapidly over the last two decades. As shown in the protein-protein network diagram (**Figures 1C-E**), a complex molecular machinery is required for LG exocytosis at the IS. However, the level of complexity is lower than in neurons. One reason for the differences between LG release at the IS and synaptic transmission in neurons could be the different time scale of each process. While synaptic transmission requires millisecond precision for SV exocytosis, LGs are released within minutes after IS formation. Thus, many proteins required to halt SV release and maintain SVs in a readily releasable state are probably not required in CTLs. In addition, the transient nature of the IS itself prevents unwanted LG release and controls the timing of exocytosis.

### 4.1 Requirement for docking/priming in LG exocytosis

**Figure 2:**
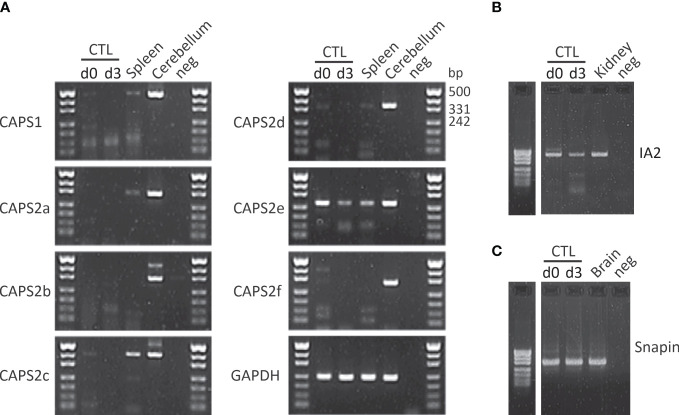
CAPS2e, IA2 and snapin mRNA are found in mouse CD8+ T cells. **(A)** RT-PCR of murine naïve (d0) and day3 (d3) stimulated CTLs and spleen using primers specific for CAPS1 and all known CAPS2 splice variant as described in Nguyen Truong et al. (2014). Total RNA isolated from cerebellum was used as positive control, water was used as negative control and the housekeeping gene *GAPDH* as loading control. Note that only CAPS2e was detected in CTL and spleen. Data are representative of two independent experiments from two mice. **(B, C)** CTL mRNA expression profile of IA2 **(B)** and snapin **(C)** before and after 3 days of stimulation with anti-CD3/CD28 antibody-coated beads (d3). Unstimulated CTLs (d0) were harvested directly after CTLs isolation. Total RNA of adult mouse brain and kidney were used as positive control for snapin and IA2, respectively and H_2_O was used as the negative control for PCR. Data are representative of two independent experiments from two mice. See supplementary material file for materials and methods.

As indicated above release of neurotransmitter requires a coordinated sequence of tethering and docking/priming factor interactions. Until now Munc18-2 and Munc13-4 have been identified in CTLs. To date no systematic study of other docking/priming factor expression has been performed in CTLs. We detected several additional putative docking/priming in CTL at the mRNA level (**Figure 2**). CAPS2e, appears to be the only CAPS isoform expressed in activated CTLs (**Figure 2A**). When examining CAPS2e function, CAPS2 knock-out CTLs did not show any changes in lysosomal compartment degranulation (data not shown). Since LGs are a subpopulation of lysosomes, presumably CAPS2e does not contribute to LG exocytosis. We further detected DOC2B mRNA in CTLs. This protein is of particular interest as it has been identified as a priming factor for lysosome secretion in mast cells (166). It is possible that it plays a similar function in CTLs. We also found mRNA of the transmembrane protein IA2 and the SNARE associated protein snapin at the mRNA level but have not investigated their function in CTLs (**Figures 2B, C**). Snapin is an interesting candidate as it has been shown to interact with SNAP23 (167), but it could be involved in lysosome recycling as well (168).

Whether LG exocytosis requires other docking/priming factors is not known. We speculate that this is not the case for 3 reasons. 1. The exocytosis process in CTLs is relatively slow. 2-3 minutes are needed between the time of IS formation and the release of the first LGs (105, 158). An ultra-rapid release of lytic granules has been described in human CTL as a key molecular mechanism of multiple target cell killing (149, 169). However, in this context lytic granule secretion, that precedes microtubule re-organization (123, 149), occurs within tens of second after CTL/target cell contact, a time frame still delayed when compared to neuronal synaptic transmission. 2. The timing of LG exocytosis is dictated by IS formation and the subsequent polarized accumulation of t-SNAREs at the plasma membrane (135, 136). 3. None of the individual vesicle pools have been detected in CTLs (117, 170). Therefore, presumably no protein is required to halt or maintain LGs in an intermediate release-ready state as is the case in neurons. As a consequence, proteins such as complexin or tomosyn might be unnecessary in this context.

### 4.2 Specific molecular players for LG release

An intriguing point is that LG exocytosis involves proteins with a function that is not required in neuron synapses. One example is Vti1b. This SNARE protein is involved in endosomal fusion events (171–173). Qu and colleagues demonstrated that docking of LG at the IS, requires tethering of LG with CD3-containing endosomes *via* Vti1b in human CTLs through an unknown interaction partner (174) (**Figure 1D**). They showed that in comparison to untethered LGs, LG tethering increased their dwell time at the IS and their release probability. Accordingly, downregulation of Vti1b reduced LG tethering, their docking at the IS, and target cell killing. However, Vti1b does not seem to directly mediate the final fusion step of LGs. It plays a role upstream of fusion, clearly affecting CTL cytotoxicity (174, 175). This is reminiscent of the function of VAMP8 or syntaxin7 in CTL (116, 136, 176).

Our interaction diagram clearly shows that many open questions remain (**Figure 1**). For example: is an interaction between CRAC channels and t-SNAREs required for perfect LG positioning prior to exocytosis? What is the Ca^2+^ sensor for LG exocytosis? What are the interaction partners for Vti1b? Systematic analysis of protein-protein interactions with pull-down assays or lipid mixing assays should be applied to CTLs (120) to shed light on these and other outstanding questions.

### 4.3 Calcium signaling

Ca^2+^ signaling differs significantly between the two synaptic types. First, different Ca^2+^ channels are involved. While in neuronal synapse Ca^2+^ enters the presynapse through VGCCs that inactivate relatively rapidly, in the immunological synapse Ca^2+^ permeates the plasma membrane *via* CRAC channel complexes which do not inactivate thanks to the buffering function of mitochondria (see section 3.3). This leads to distinct intracellular Ca^2+^ concentration ([Ca^2+^]_i_) increases. Stimulation of a neuronal synapse by one action potential induces a very short (> 10 ms), extremely localized (1-2 µm diameter) but steep (> 10 µM) [Ca^2+^]_i_ increase, which is sufficient for the fusion of one to three SVs (177, 178). In addition, a typical action potential train at 10 Hz elicits a prolonged Ca^2+^ influx that phases out after a few seconds due to inactivation of VGCCs. The ensuing [Ca^2+^]_i_ increase spreads from the active zone to the back of the synapse. In contrast, stimulation of CTL by a target cell induces a biphasic [Ca^2+^]_i_ increase. First IS formation induces an IP_3_ mediated Ca^2+^ release from the endoplasmic reticulum raising the [Ca^2+^]_i_ transiently. The Ca^2+^ concentration depletion in the endoplasmic reticulum then activates the CRAC channel complex causing a long lasting (minutes to hours) increases in [Ca^2+^]_i_ (see section 3.3). The rise of [Ca^2+^]_i_ is relatively uniform along the IS and can reach values of about 2 µM (133). The Ca^2+^ then spreads throughout the cytoplasm of the CTL. Some functions of Ca^2+^ are conserved in both cell types. In neurons it is undisputed that Ca^2+^ triggers the fusion of SV with the plasma membrane. Although this function is still under debate in CTLs, a large body of work indicate that this is also the case (see Kaschek et al. (179) for review). Additionally, Ca^2+^ promotes endocytosis allowing a tight coupling between exo- and endocytosis in both cell types. Other functions such as regulating cytoskeleton remodeling or vesicle transport are probably different.

### 4.4 Variability of LG ultrastructure and composition

The diversity of secreted organelles is an important difference between neuronal cells and CTL secretion. Neurons secrete SVs at synapses and large dense core vesicles (LDCVs) along the entire plasma membrane. Both organelles have well-defined shapes and sizes. The release machinery of LDCV is not fully understood, but appears to be very similar to that of SV exocytosis with some differences in docking/priming factors (180, 181). This is very different for CTLs. Not only do they release very different types of organelles *via* regulated exocytosis at the IS (Rab11-positive vesicles, LGs and MVBs), but even the LGs are diverse. As mentioned above, LGs can be divided into single-core granules, containing diffusible granzyme B, and multi-core granules, containing SMAPs (119, 154). The SCGs have a well-defined round shape with a diameter of 293 ± 8 nm, whereas the MCG are spheres with a more or less elongated shape of and their size is quite variable with an average diameter of 364 ± 12 nm. These shape and size differences could affect their fusion kinetics (182, 183). Furthermore, it is completely unclear whether they are released in parallel, at different times after IS formation, or upon specific stimuli. If the latter is the case, then different tethering or docking/priming factors should control the exocytosis of single *vs.* multi-core granules. Understanding the specificity of different LG release would be particularly important for fine-tuning the duration and intensity of lytic function of killer cells (119, 154).

## 5 Concluding remarks

Both the nervous and immune systems use synaptic contacts to transmit key intercellular information through regulated secretion of intracellular vesicles. They use a similar molecular machinery, with SNAREs at the core and Munc18 and Munc13 as the major facilitators. The protein paralogs in CTLs are different from those in neurons, but their individual domain structures are nearly identical, with the exception of Munc13. Thus, advancing knowledge of exocytosis in one system benefits the study of the other.

A great advantage of studying secretion in immune synapses over neuronal synapse is that human CTL are readily available and can be easily handled. Accumulated research findings in CTL reveal subtle differences in the mouse and human T cell secretory machinery. One example for this diversity is the v-SNARE of LG that is required for exocytosis: VAMP7 is used in human CTL, while VAMP2/synaptobrevin2 is used by mouse CTL (118, 142). VAMP7 is also expressed in mouse CTLs but its function remains to be determined. Similar interspecies differences have not yet been identified for synaptic transmission in the nervous system probably because human neurons are nearly inaccessible. This might change in the near future as human neurons can now be derived from induced pluripotent stem cells (184, 185). In the meantime research performed on human CTLs might be instrumental to shed new light on human neuronal synapses. Understanding interspecies differences is especially important when studying the effect of mutations like those found in FHL. Until now, mutations in Munc13-4, Munc18-2, Rab27a, syntaxin 11, SNAP23 have been regarded as knock out or knock down phenotype. However, the situation might be more complex as subtle alterations in the function of these proteins might contribute to multiple shades of synaptic transmission in immune cells and neurons. Other advantages of working with immune cells is the easier molecular manipulation of blood cells as opposed to nervous system tissue and the possibility to inspect lymphocytes using a panel of high-resolution imaging techniques difficult to apply to human neurons.

In conclusion, in our review, we discussed how “synaptic inspection” in neuroscience and immunology can learn from each other and how important it is to define the precise identity and function of the multiple proteins involved in both SV and LG secretion.

## Author contributions

UB, H-FC, and CS conceived and wrote most of the paper. VP and EK wrote some parts of the paper. CS and VP generated the data shown **Figure 2**. UB prepared the figures. All authors contributed to the article and approved the submitted version.
