# Supplementary material for: Lytic granule exocytosis at immune synapses: lessons from neuronal synapses
Source: Front Immunol. 2023 May 18;14:1177670. doi: 10.3389/fimmu.2023.1177670 (PMC10233144; doi:10.3389/fimmu.2023.1177670)
Supplement: Supplementary file 1 [file DataSheet_1.docx]

**Supplementary materials and methods**

**Mice.**

All experimental procedures were approved and performed according to the regulations by the state of Saarland (Landesamt für Verbraucherschutz, AZ.: 2.4.1.1).

**CTL cell culture**

Splenocytes were isolated from spleens of WT mice. Spleens were smashed through 70 µm cell strainer in cold RPMI medium supplemented with 10% FCS and 1% Penicillin/Streptomycin (P/S). Splenocytes were then collected after removing erythrocytes from cell suspension by water-based lysis buffer that contained 155 mM NH_4_Cl, 10 mM KHCO_3_ and 0.13 mM EDTA. 1 ml lysis buffer was applied to the splenocyte pellet from one spleen for 30 seconds at RT. Afterwards, 9 ml RPMI medium was supplied to the 15 ml falcon to stop the lysis reaction. After centrifuging the cells at 1200 rpm, 6 min, the cell suspension was further washed once with D-PBS containing 2 mM EDTA and 0.1% BSA before CD8 positive isolation. Naive CD8+ T cells were positively isolated from splenocytes using Dynabeads FlowComp Mouse CD8+ kit (Invitrogen) as described by the manufacturer. The isolated naive CD8+ T cells were stimulated with anti-CD3/anti-CD28 activator dynabeads (1:0.8 ratio; Invitrogen) and cultured for 5 days. Cells were cultured at a density of 1×10^6^ cells/ml in 24-well culture plate (2 million cells per well) with AIMV medium (Invitrogen) containing 10% FCS, 1% P/S (Invitrogen) and 50 μM 2-mercaptoethanol (BME). 50 U/ml recombinant mouse IL-2 (Gibco) was applied in the first 2 days of T cell culture. Afterwards, 100 U/ml mouse IL-2 was given after 48 h of culture to support T cell proliferation.

**RT-PCR**

Total RNA was isolated from homogenized tissue or cells by TRIzol (Thermo Fisher Scientific) preparation reverse transcribed with SuperScript^TM^ (Thermo Fisher Scientific) using hexamer random primers. Semi-quantitative PCR was performed using intron-spanning primers and 10 ng cDNA. Primers for CAPS1, CAPS2 splice variants and *GAPDH* were used as described in Nguyen Truong et al. (2014). For Snapin and IA2 we used the following primers: Snapin-fwd (5’ gctggttccgctgctgtgtca 3’), Snapin-rev (5’ tcgttcctgtgcattctgtaa 3’), IA2-fwd (5’ ggcccatggtga cactacttttga 3’) and IA2-rev (5’ tcgatgatggggctggcgttgatg 3’) to amplify a 306 bp and 459 bp PCR products, respectively.

**References**

Nguyen Truong, C.Q., Nestvogel, D., Ratai, O., Schirra, C., Stevens, D.R., Brose, N., et al. (2014). Secretory vesicle priming by CAPS is independent of its SNARE-binding MUN domain. *Cell Rep* 9(3)**,** 902-909. doi: 10.1016/j.celrep.2014.09.050.
